# CTC1 increases the radioresistance of human melanoma cells by inhibiting telomere shortening and apoptosis

**DOI:** 10.3892/ijmm.2014.1721

**Published:** 2014-04-02

**Authors:** Y.M. LUO, N.X. XIA, L. YANG, Z. LI, H. YANG, H.J. YU, Y. LIU, H. LEI, F.X. ZHOU, C.H. XIE, Y.F. ZHOU

**Affiliations:** 1 Hubei Cancer Clinical Study Center, Hubei Key Laboratory of Tumor Biological Behaviors, Wuhan University, Wuhan, Hubei, P.R. China; 2 Department of Radiation Oncology and Medical Oncology, Zhongnan Hospital, Wuhan University, Wuhan, Hubei, P.R. China

**Keywords:** telomere-binding protein, conserved telomere maintenance component 1, radioresistance

## Abstract

Melanoma has traditionally been viewed as a radioresistant cancer. However, recent studies suggest that under certain clinical circumstances, radiotherapy may play a significant role in the treatment of melanoma. Previous studies have demonstrated that telomere length is a hallmark of radiosensitivity. The newly discovered mammalian CTC1-STN1-TEN1 (CST) complex has been demonstrated to be an important telomere maintenance factor. In this study, by establishing a radiosensitive/radioresistant human melanoma cell model, MDA-MB-435/MDA-MB-435R, we aimed to investigate the association of CTC1 expression with radiosensitivity in human melanoma cell lines, and to elucidate the possible underlying mechanisms. We found that CTC1 mRNA and protein levels were markedly increased in the MDA-MB-435R cells compared with the MDA-MB-435 cells. Moreover, the downregulation of CTC1 enhanced radiosensitivity, induced DNA damage and promoted telomere shortening and apoptosis in both cell lines. Taken together, our findings suggest that CTC1 increases the radioresistance of human melanoma cells by inhibiting telomere shortening and apoptosis. Thus, CTC1 may be an attractive target gene for the treatment of human melanoma.

## Introduction

Melanoma is an extremely aggressive cancer with a high metastatic potential and is responsible for the majority of skin cancer-related mortality (1). It is now the 5th and 6th leading cause of cancer in men and women, respectively (2). For several years, melanoma has been considered to be a highly radioresistant tumor due to its efficient DNA repair mechanisms (3). However, a number of studies have suggested that melanoma cells are heterogeneous and hence should not be generally considered radioresistant (4–7). Finding agents that sensitize malignant cells to radiation would augment tumor response, while minimizing toxicity to surrounding normal tissues. A number of extranuclear and intranuclear factors have been identified to influence the radiation responsiveness of melanoma cells, including breast cancer 1, early onset (BRCA1), excision repair cross-complementing rodent repair deficiency, complementation group 1 (ERCC1), poly(ADP-ribose) polymerase (PARP), transglutaminase 2 (TGM2) and SLUG, as well as chromosomal characteristics, such as telomere length (8,9).

Telomeres, the specialized structures located at the chromosome terminus, play important roles in the cellular response to DNA damage and chromosomal stability. They consist of telomeric DNA and a suite of telomere-binding proteins (TBPs), both of which are responsible for telomere function. Six TBPs forming shelterin have been reported as the telomeric core complex, telomeric repeat-binding factor 1 and 2 (TRF1 and 2), protection of telomeres 1 (POT1), repressor/activator protein 1 (RAP1), TRF1-interacting nuclear protein 2 (TIN2), and tripeptidyl peptidase I (TPP1; formerly known as TINT1, PTOP, or PIP1). They play essential roles in the regulation of telomere length by inhibiting excessive nuclease activity at the chromosome ends and by regulating telomerase, the enzyme that elongates telomeres (10–13). Other TBPs include Ku70/80, the MRN complex (MRE11/Rad50/Nbs1), DNA-PKcs and tankyrase 1/2.

The newly identified mammalian conserved telomere maintenance component 1 (CTC1)-STN1-TEN1 (CST) complex binding to the telomeres has also been demonstrated to promote telomere integrity (14). Mammalian STN1 and TEN1 are the sequence homologues of budding and fission yeast proteins (14–16). The third member of the complex, CTC1, is not a sequence homologue of Cdc13, although it shares functional similarities. In addition, CTC1 binds to the telomeric single-stranded DNA (ssDNA) in a sequence-independent manner together with STN1 and TEN1 (14). A genome-wide meta-analysis has pointed to CTC1 as a gene regulating telomere homeostasis in humans (17). The depletion of human CTC1 by RNAi *in vitro* triggers a DNA damage response, chromatin bridges, the accumulation of G-overhangs, the inhibition of telomerase and sporadic telomere loss (18,19). However, the role of human CTC1 in the response of melanoma cells to ionizing radiation remains unknown. In this study, we established a radiosensitive/radioresistant human melanoma cell model, MDA-MB-435/MDA-MB-435R, in order to investigate the mechanisms responsible for radioresistance. Our data demonstrate that CTC1 expression is markedly decreased in the radiosensitive melanoma cells compared with the radioresistant cells. Moreover, the knockdown of CTC1 imparts radiosensitivity to human melanoma cells by enhancing telomere shortening and inducing cell apoptosis.

## Materials and methods

### Cell culture and transfection

The human melanoma cell line, MDA-MB-435, was purchased from the Cell Bank of the Chinese Academy of Sciences, Shanghai, China. The relative radioresistant cell line, MDA-MB-435R, was established in our laboratory by the repeated irradiation of MDA-MB-435 cells (unpublished data). Irradiation, 6 MV X-ray, was produced by a linear accelerator (Siemens, Munich, Germany) at a dose rate of 2 Gy/min. The D_0_ value of the MDA-MB-435R cells (3.266±0.072) markeldy increased significantly compared to that of the MDA-MB-435 cells (2.093±0.131).

The cells were maintained in Roswell Park Memorial Institute (RPMI)-1640 medium (Life Technologies, Grand Island, NY, USA) supplemented with 10% heat-inactivated fetal bovine serum, 100 U/ml penicillin and streptomycin (Life Technologies) at 37°C in a humidified atmosphere of 5% CO_2_.

siRNA was designed against human CTC1 (GenBank accession no. NM_025099.5) with the following sequences: 5′-CCAGAUCUCACAAUGUUUATT-3′ synthesized by GenePharma (Shanghai, China). The sequence, 5′-GTTCTCC GAACGTGTCACGT-3′, was used as the non-silencing control (negative control) in all the experiments. Transfection was performed using Lipofectamine 2000 (Invitrogen, Carlsbad, CA, USA) according to the manufacturer’s instructions. Cells transfected with siCTC1#1–3, non-silencing siRNA and transfection reagents alone were termed the siCTC1#1–3, siNC and mock group, respectively. Cells without any treatment were used as the untreated group. At 48 h post-transfection, the cells were harvested for the following assays.

### RNA extraction and quantitative reverse transcription PCR (qRT-PCR)

Total RNA was isolated using TRIzol reagent (Invitrogen) following the manufacturer’s instructions. First-strand cDNA was obtained using the RevertAid™ First-Strand cDNA Synthesis kit (Fermentas International, Inc., Burlington, ON, Canada). For the quantitative analysis of CTC1 mRNA, the human GAPDH gene was used as an internal control. Primer sequences were designed as follows: CTC1 sense, 5′-TGGACC TTTCTTGGTTGGG-3′ and antisense, 5′-AGGACAGGGAT AGGCGTGA-3′; GAPDH sense, 5′-ATCACTGCCACCCAG AAGAC-3′ and antisense, 5′-AGCGTCAAAGGTGGAGG AGT-3′. The resulting cDNA was detected using SYBR-Green PCR Master mix (Takara, Shiga, Japan) with Mx3000P (Stratagene, La Jolla, CA, USA). For the measurements of relative telomere length, the single copy gene, 36B4 (encodes acidic ribosomal phosphoprotein), was used as an endogenous control. The primers used for the telomeres were as follows: sense, 5′-GTTTTTGAGGGTGAGGGTGAGGGTGAGGG TGAGGGT -3′ and antisense, 5′-TCCCGACTATCCCTAT CCCTATCCCTATCCCTATCCCTA-3′. The primers used for 36B4 were sense, 5′-CAGCAAGTGGGAA GGTGTAATCC-3′ and amtisense, 5′-CCCATTCTATCATC AACGGGTACAA-3′. The Mx3000P analysis program was used to analyze the results.

### Western blot analysis

Cells in a 100-mm dish were rinsed twice with cold phosphate-buffered saline (PBS), and lysed with 200 μl radio immunoprecipitation assay (RIPA) lysis buffer and 1 mM PMSF (Beyotime Institute of Biotechnology, Shanghai, China). A scraper was used to remove the cells into an Eppendorf tube. After the cells were ultrasonicated and centrifuged at 12,000 rpm for 10 min, the supernatant was collected and incubated in boiled water for 3–5 min. Protein concentration was determined using a BCA protein assay kit (Beyotime Institute of Biotechnology). Protein extracts (50 μg) were electrophoresed on 8% SDS-PAGE gels and then transferred on to PVDF membranes. The blots were blocked for 1 h at room temperature in 5% non-fat milk in Tris-buffered saline with 0.1% Tween-20, and incubated at 4°C overnight with the following antibodies: anti-CTC1 and anti-GAPDH (Santa Cruz Biotechnology, Inc., Santa Cruz, CA, USA). After washing and incubating with secondary antibodies, the bands were visualized using the ECL Plus kit (Beyotime Institute of Biotechnology) and recorded onto X-Omat AR film (Eastman Kodak Co., Rochester, NY, USA). The density of each band was quantified using ImageJ software.

### Clonogenic survival assay

Teh cells were plated onto 6-well plates with different cell numbers (100–2,000) for each dose group overnight and subjected to 6 MV X-ray at the dose of 0–10 Gy as indicated. After 14 days of incubation, the colonies were fixed and stained with 1% crystal violet in absolute alcohol. The surviving colonies (≥50 cells/colony) were scored while being viewed under an inverted phase contrast microscope (Olympus, Tokyo, Japan). A single-hit multi-target model (20) was used to analyze the data. The survival curve of each group was plotted as the log of the survival fraction vs. the radiation dose using GraphPad Prism 5.0 software.

### Immunofluorescence assay by confocal laser scanning microscopy

The immunofluorescence detection of γH2AX foci was used to determine the residual DNA double-strand breaks (DSBs). Cells grown on round coverslips (Fisher Scientific, Hampton, NH, USA) were divided into 4 groups: siCTC1#3, ionizing radiation (IR), siCTC1#3 + IR and the untreated group. The IR and siCTC1#3 + IR cell groups were exposed to 6 Gy X-ray at 36 h post-transfection. Twelve hours later, the cells were fixed in ice-cold 4% formaldehyde for 20 min, treated with 0.2% Triton X-100 for 15 min, blocked in 5% (w/v) BSA for 1 h and incubated with antibody to γH2AX (ser139, dilution 1:400; Millipore Corp., Billerica, MA, USA) overnight at 4°C. After washing with PBS, the secondary FITC-conjugated antibody (Millipore Corp.) was added for 45 min at 37°C, and the slides were washed with PBS and stained with DAPI. Subsequently, images were recorded using a confocal microscope (Leica Microsystems GmbH, Wetzlar, Germany). The percentage of γH2AX foci-positive cells and the number of γH2AX foci per cell were determined by analyzing 100 randomly selected cells.

### Flow cytometric analysis of apoptosis

For apoptosis assay, the cells were divided into 6 groups: siCTC1#3, negative control siRNA (siNC), IR, siCTC1#3 + IR, siNC + IR and the untreated group. Cells in the IR, siCTC1#3 + IR and siNC + IR groups were exposed to 6 Gy X-ray at 24 h post-transfection. Another 24 h later, both Anexin V-fluorescein isothiocyanate and propidium iodide (PI) were used to stain the cells according to manufacturer’s instructions (Beyotime Institute of Biotechnology). The occurrence of apoptosis was quantified by flow cytometry (Cytomics FC 500; Beckman Coulter, Fullerton, CA, USA).

### Statistical analysis

All the experiments were repeated at least 3 times. Data are presented as the means ± standard deviation (SD). The quantification of band density was performed using ImageJ software. Statistical analysis was performed with one way ANOVA using SPSS 18.0 and GraphPad Prism 5.0 software. A value of P<0.05 was considered to indicate a statistically significant difference.

## Results

### siRNA against CTC1 effectively reduces CTC1 mRNA and protein expression

**Figure 1:**
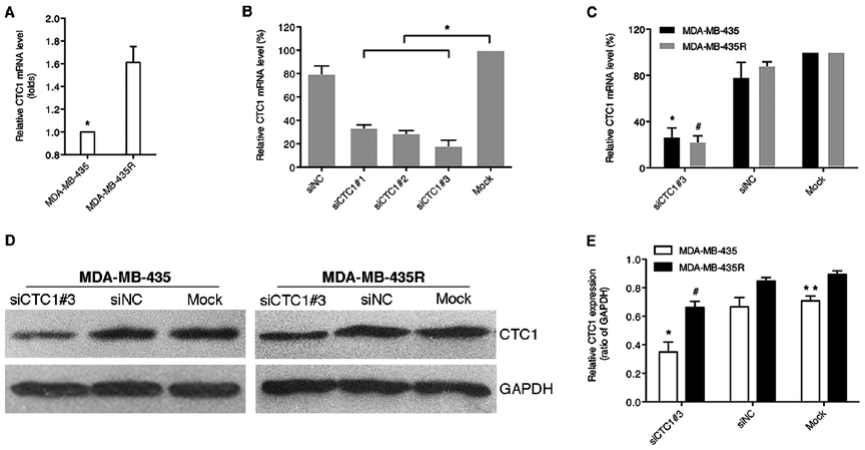
siRNA against CTC1 decreases CTC1 mRNA and protein levels. (A) Quantitative PCR detected the relative CTC1 mRNA level in the MDA-MB-435 and MDA-MB-435R cells. (B) MDA-MB-435 cells were transfected with siCTC1#1–3, siNC or mock siRNA. (C) Relative CTC1 mRNA level alterations in MDA-MB-435 and MDA-MB-435R cells transfected with siCTC1#3, siNC or mock. The knockdown percentages are relative to the mock transfection group, which was set at 100%. (D) Western blot analysis showed the relative protein levels of CTC1 in MDA-MB-435 and MDA-MB-435R cells transfected with siCTC1#3, siNC or mock. (E) Bar chart shows the semi-quantitative analysis of CTC1 protein levels. The bar graph shows the mean ± standard deviation (SD) values of 3 independent experiments. ^*^P<0.05 and ^#^P<0.05 vs. siNC and mock group, respectively; ^**^P<0.05 vs. mock group of MDA-MB-435 cells. siNC groups revealed no apparent differences with the mock groups.

qRT-PCR and western blot analysis were used to analyze the altered relative mRNA and protein expression of CTC1. The MDA-MB-435R cells showed higher CTC1 mRNA and protein expression levels compared with the MDA-MB-435 cells (Fig. 1A). Three siRNAs against CTC1 effectively reduced the relative mRNA levels of CTC1 in the MDA-MB-435 cells (P<0.05), among which siCTC1#3 was the most effective (Fig. 1B). In contrast to the siNC and mock group, the relative CTC1 mRNA and protein levels of both cell lines were markedly decreased in siCTC1#3 group (P<0.05), while the control groups showed no apparent changes (Fig. 1C–E). These results indicated that siCTC1#3 effectively suppressed the CTC1 mRNA and protein levels in the MDA-MB-435 and MDA-MB-435R cells.

### Downregulation of CTC1 sensitizes cells to radiation

**Figure 2:**
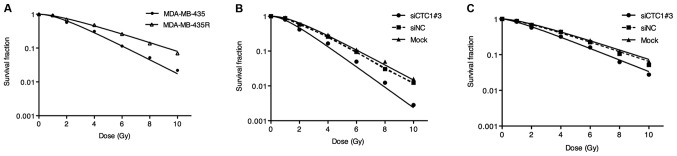
Downregulation of CTC1 sensitizes melanoma cancer cells to radiation. (A) Survival curves of untreated MDA-MB-435 and MDA-MB-435R cells. Survival curves of (B) MDA-MB-435 and (C) MDA-MB-435R cells transfected with siCTC1#3, siNC or mock. Each group of cells was irradiated at the dose point of 0, 1, 2, 4, 6, 8, 10 Gy. At 14 days post-incubation, the colonies were fixed and stained. The surviving colonies (≥50 cells/colony) were scored. The data were fit into the single-hit multi-target model, and survival curves were demonstrated using GraphPad Prism 5.0 software.

In order to investigate the role of CTC1 in modulating the radiosensitivity of the MDA-MB-435 and MDA-MB-435R cells, CTC1 was downregulated by siRNA. The survival curves described the radiobiological parameters of each group. As shown in Fig. 2A, the survival fractions of the MDA-MB-435R cells increased significantly at the 2, 4, 6, 8 and 10 Gy dose point compared with those of the MDA-MB-435 cells (P<0.05). Compared to the siNC and mock group, the survival fractions of the siCTC1#3 group markedly decreased at the 2, 4, 6, 8 and 10 Gy dose point (Fig. 2B and C). The radiobiological parameters calculated according to the curves are presented in Table I. The D_0_, Dq and SF2 values in the cells transfected with siCTC1#3 were significantly lower than the siNC and mock groups in both cell lines (P<0.05), while the siNC groups showed no significant differences with the mock groups. We concluded that the downregulation of CTC1 enhances the radiosensitivity of MDA-MB-435 and MDA-MB-435R cells.

### CTC1 increases the γH2AX-mediated repair of DNA DSBs

**Figure 3:**
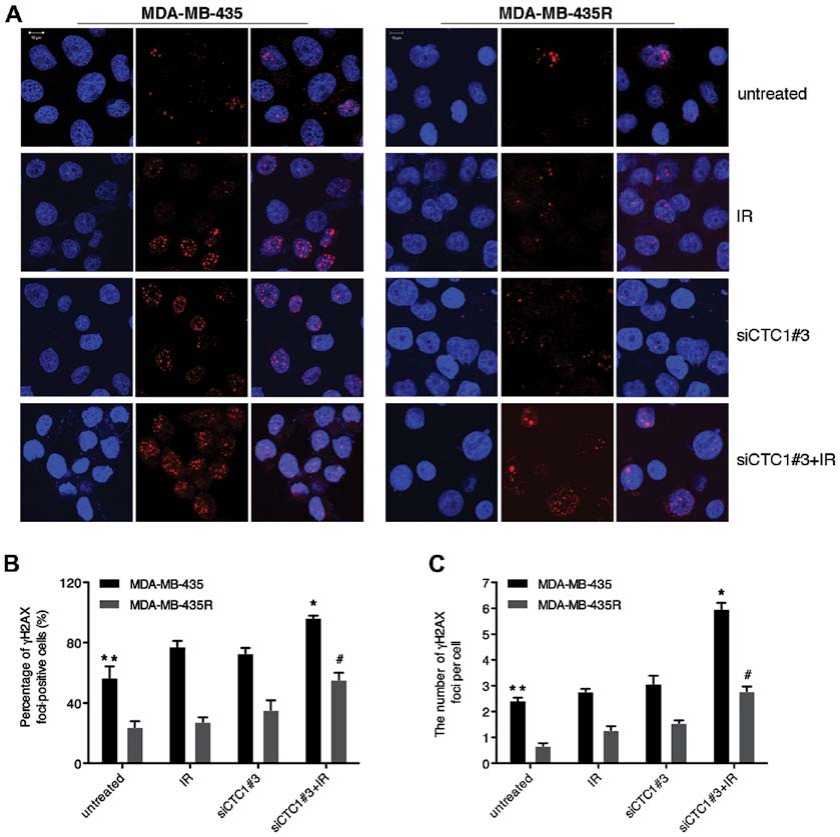
CTC1 increases the γH2AX-mediated repair of DNA double-strand breaks (DSBs). Cells were harvested 12 h post-ionizing radiation (IR). (A) Images were recorded using confocal microscopy. Nuclei were stained with DAPI (blue) and antibody to H2AX (red). (B) The percentage of γH2AX foci-positive cells and (C) the number of γH2AX foci per cell (C) were determined by analyzing 100 randomly selected cells. Size marker, 10 μm. Bar graph shows the mean ± standard deviation (SD) values of 3 independent experiments. ^**^P<0.05 vs. untreated MDA-MB-435R cells; ^*^P<0.05 and ^#^P<0.05 vs. IR, siCTC1#3 or untreated group in each cell line.

As a histone H2A variant, H2AX plays an essential role in the cellular response to DNA DSBs. H2AX senses DSBs through rapid serine 139 phosphorylation, and forms phospho-γH2AX foci with various proteins. In the cells with different sensitivity to IR-induced DSBs, γH2AX selectively recruites specific proteins to decide different cell fates (21). As shown in Fig. 3, γH2AX foci in the MDA-MB-435R cells significantly decreased compared with the MDA-MB-435 cells (P<0.05). Treatment with siCTC1#3 led to increased IR-induced γH2AX foci in both cell lines (P<0.05). Our data indicate that CTC1 is actively involved in repairing DSBs to reduce γH2AX foci in MDA-MB-435 and MDA-MB-435R cells.

### Depletion of CTC1 causes telomere length attrition

**Figure 4:**
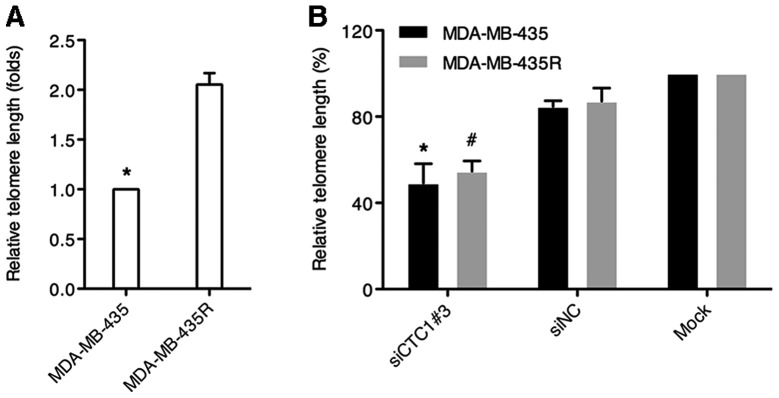
Depletion of CTC1 causes telomere length attrition. Quantitative PCR detected the relative telomere length in (A) MDA-MB-435 and MDA-MB-435R cells and (B) cells transfected with siCTC1#3, siNC or mock. The bar graph shows the mean ± standard deviation (SD) values of 3 independent experiments. ^*^P<0.05 and ^#^P<0.05 vs. siNC and mock groups, respectively. siNC groups revealed no apparent differences with the mock groups.

Relative telomere lengths were determined by quantitative PCR. The dissociation curves showed a unique peak from the PCR amplification of the telomeres and the single copy gene, 36B4. Our data demonstrated that the relative telomere length in the MDA-MB-435R cells was almost twice that in the MDA-MB-435 cells (P<0.05) (Fig. 4A). As shown in Fig. 4B, the cells (both cell lines) transfected with siCTC1#3 exhibited obvious telomere shortening compared with the cells transfected with siNC or the mock-transfected cells (P<0.05). These results suggest that CTC1 is involved in telomere maintenance in MDA-MB-435 and MDA-MB-435R cells.

### Combination of siCTC1 and radiation increases cell apoptosis

**Figure 5:**
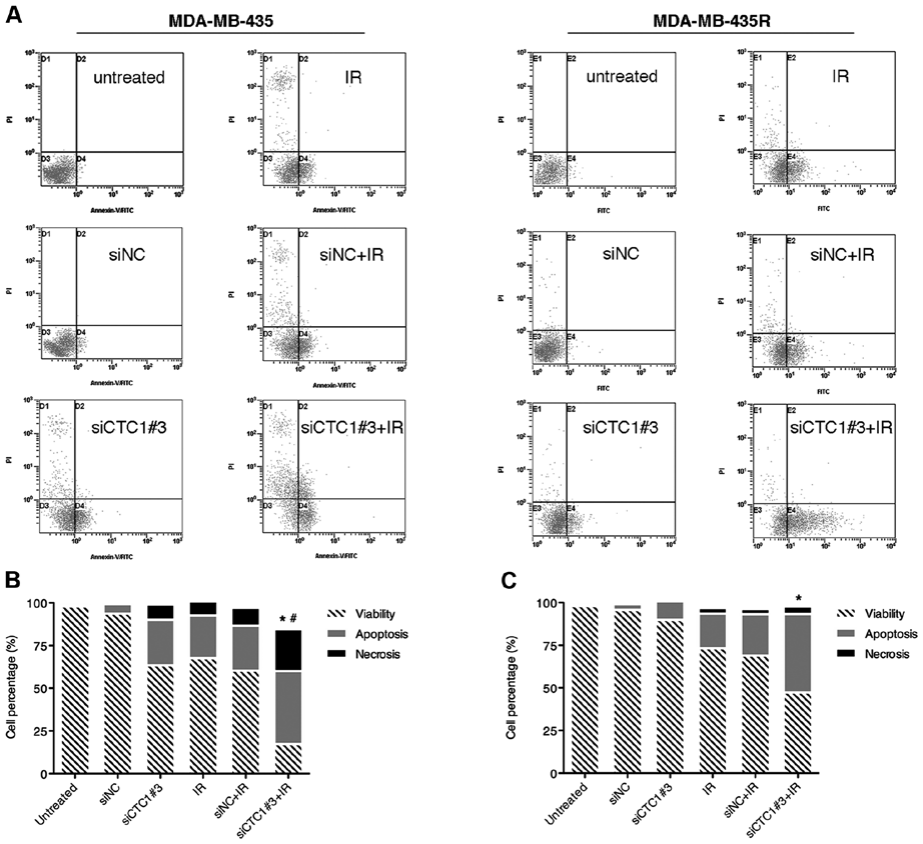
Combination of siCTC1 and radiation increases cell apoptosis. (A) Flow cytometry was used to determine the effects of siCTC1 on cell death prior to and post-irradiation in MDA-MB-435 and MDA-MB-435R cells. Cells were stained with Annexin V-fluorescein isothiocyanate and propidium iodide (PI) and classified into 4 subpopulations: viable cells (Annexin V and PI double-negative), apoptotic cells (Annexin V-positive), early dead cells (Annexin V and PI double-positive) and dead cells (PI=positive). Percentages of cell apoptosis, necrosis and viability in (B) MDA-MB-435 and (C) MDA-MB-435R cells were calculated. Data are presented as the mean values of 3 independent experiments. ^*^P<0.05 vs. ionizing radiation (IR) or siCTC1#3 (representing differences in apoptotic rates), ^#^P<0.05 vs. IR or siCTC1#3 (representing differences in necrosis rate).

It is known that irradiation can cause DNA damage. If DNA damage is not adquately repaired after irradiation, cells may progress towards apoptosis and/or necrosis. Since siRNA against CTC1 sensitized the MDA-MB-435 and MDA-MB-435R cells to irradiation, we further investigated the effects of siCTC1 on cell death post-irradiation. Twenty-four hours after irradiation, the cells were harvested and stained with Annexin V-fluorescein isothiocyanate and PI and classified into 4 subpopulations as follows: viable cells (Annexin V and PI double-negative), apoptotic cells (Annexin V-positive), early dead cells (Annexin V and PI double-positive) and dead cells (PI-positive). The results (Fig. 5) revealed that while the single treatment with siCTC1#3 or IR increased the apoptotic rates in both cell lines, the combined treatment produced an even greater number of apoptotic cells (P<0.05). Moreover, the combined treatment of MDA-MB-435 cells significantly increased the rate of necrosis compared to the groups with the single treatment (P<0.05). siCTC1, in conjunction with IR, enhances the killing effect on MDA-MB-435 and MDA-MB-435R cells.

## Discussion

In this study, to the best of our knowledge, we demonstrate for the first time that CTC1 expression is associated with radioresistance in human melanoma cells.

Although melanoma is a relatively radioresistant tumor type, our greater radiobiological understandings may provide better control for certain clinical situations. In order to improve the therapeutic ratio, there has been increasing interest in studying the difference between radioresistant cells and their parental counterparts. The cell lines used in this study were derived from the same origin, which may have similar tumor characteristics. Thus, this may be a good model to investigate determinant factors for radiosensitivity. Our results demonstrated that the MDA-MB-435 cells were more radiosensitive than the MDA-MB-435R cells and that the knockdown of CTC1 increased the radiosensitivity of both cell lines (Fig. 2). Thus, we infer that the knockdown of CTC1 may act as a potent radiosensitizer in human melanoma cell lines.

Telomere maintenance has been implicated in ageing and cancer, and requires the cooperation of a multitude of telomeric proteins. Some of these proteins are associated exclusively with telomeres, whereas others localize to additional subnuclear or subcellular sites (22). The altered expression of telomeric proteins may disrupt the capping complex, facilitating telomere degradation and shortening, regardless of the telomerase status (23,24). The prevailing view has been that two distinct telomeric capping complexes evolved, shelterin in vertebrates and a trimeric complex termed CST (Cdc13, STN1 and TEN1) in yeast. However, the recently discovered CST-like complex in plants and humans raises new questions as to the composition of telomeres and their regulatory mechanisms in multicellular eukaryotes (14,15,18). Genetic data argue that CST and shelterin act in distinct pathways to maintain telomere integrity in human cells. The significant increase in the frequency of telomere dysfunction-induced foci (TIF) in STN1/POT1-knockdown cells compared to the STN1 or POT1 single knockdown cells, suggests that CST and POT1 play redundant roles in telomere protection (14). It is now widely accepted that telomere length acts as a hallmark of radiosensitivity (25–30). Consequently, we investigated whether the radiosensitization of CTC1 downregulation was the result of telomere length attrition. As expected, our data revealed that telomere length in the MDA-MB-435R cells was almost twice that in the MDA-MB-435 cells. The knocking down of CTC1 significantly decreased telomere length in both cell lines (Fig. 4).

Recent studies have revealed that CTC1 null mice undergo a rapid onset of global cellular proliferative defects and die prematurely from complete bone marrow failure by activating an ATR-dependent G2/M checkpoint (31). Mutations in CTC1 underlie the rare human genetic disorder, Coats plus, characterized by gastrointestinal and neurological defects, as well as shortened telomeres and evidence of an ongoing DNA damage response (32). These clinical results suggest that similar to the phenotypes observed in CTC1 null mice, human CTC1 missense mutations also confer haematopoietic defects. In addition, previous studies have addressed the role of apoptosis in determining radiation response. The disabling of apoptotic responses may be a major contributor to radioresistance (33). Thus, we hypothesized that the radioresistance of MDA-MB-435R cells imparted by CTC1 may be related to the induction of apoptosis. Our results demonstrated that the knockdown of CTC1 increased the rate of apoptosis in both melanoma cell lines (Fig. 5). Overall, we know that the second mechanism responsible for CTC1 modulating radiosensitivity is through the regulation of apoptosis.

However, in our current study, the pro-apoptotic activity of anti-CTC1 in tumor cells makes it difficult to establish a stable clone to constitutively suppress CTC1. Therefore, this hindered our study of a clear-cut mechanism of CTC1 in response to radiation. In the future, we may use a Tet-on/Tet-off inducible system in stable clones, which can eliminate the problem of transfection efficiency, and may be a powerful tool for further investigation of the underlying mechanisms of CTC1 on radiation response in human melanoma cell lines.

In conclusion, the cohabitation at telomeres of the CST and shelterin components adds to the complexity of telomere biology. By establishing a radiosensitive-radioresistant human melanoma cell model, we found that CTC1 enhances the radioresistance of human melanoma cells by inhibiting telomere shortening and apoptosis. High levels of CTC1 seem to be directly associated with radioresistance in human melanoma cell lines as an independent predictive factor. Likewise, further studies on the role of CTC1 in regulating radiosensitivity in other radiosensitive-radioresistant cancer cell lines are another avenue to pursue.

## Figures and Tables

**Table I:** Radiobiological parameters in the different groups

Group	D_0_	Dq	SF2
MDA-MB-435			
Untreated	2.093±0.131^a^	2.088±0.046	0.574±0.018^a^
siCTC1#3	1.475±0.153^b^	1.472±0.095^b^	0.417±0.016^b^
siNC	1.929±0.099	2.135±0.106	0.578±0.029
Mock	1.993±0.164	2.258±0.094	0.595±0.017
MDA-MB-435R			
Untreated	3.266±0.072	2.379±0.108	0.701±0.016
siCTC1#3	2.356±0.109^c^	1.331±0.074^c^	0.565±0.031^c^
siNC	3.153±0.156	2.247±0.089	0.677±0.014
Mock	3.231±0.155	2.324±0.101	0.696±0.022

D_0_, Dq and SF2 values were calculated according to the survival curves. D_0_, the incremental dose required for reducing the fraction of colonies to 37%, indicative of single-event killing; Dq, quasi-threshold dose; SF2, the survival fraction of exponentially growing cells when irradiated at the clinically relevant dose of 2 Gy. Data are presented as the means ± SD of 3 independent experiments.

a P<0.05 vs. untreated MDA-MB-435R cells;

b P<0.05 and

c P<0.05 vs. siNC and mock group, respectively. siNC groups showed no apparent differences with the mock groups.
